# Influenza virus entry and replication inhibited by 8‐prenylnaringenin from *Citrullus lanatus* var. *citroides* (wild watermelon)

**DOI:** 10.1002/fsn3.2725

**Published:** 2022-01-23

**Authors:** Akari Hanada, Ryosuke Morimoto, Yuka Horio, Mototada Shichiri, Ayaka Nakashima, Taro Ogawa, Kengo Suzuki, Hidenobu Sumitani, Tokutaro Ogata, Yuji Isegawa

**Affiliations:** ^1^ Department of Food Sciences and Nutrition Mukogawa Women’s University Nishinomiya Japan; ^2^ Biomedical Research Institute National Institute of Advanced Industrial Science and Technology (AIST) Ikeda Japan; ^3^ Euglena, Co., Ltd Minato‐ku Japan; ^4^ Toyo Institute of Food Technology Kawanishi Japan; ^5^ Faculty of Health and Medical Sciences Hokuriku University Kanazawa Japan; ^6^ Present address: Faculty of Human Life Science Shikoku University Tokushima Japan

**Keywords:** 8‐prenylnaringenin, influenza virus, virus‐adsorption, virus‐maturation, wild watermelon

## Abstract

We previously demonstrated the anti‐influenza activity of *Citrullus lanatus* var. *citroides* (wild watermelon, WWM); however, the active ingredient was unknown. Here, we performed metabolomic analysis to evaluate the ingredients of WWM associated with antiviral activity. Many low‐molecular weight compounds were identified, with flavonoids accounting for 35% of all the compounds in WWM juice. Prenylated flavonoids accounted for 30% of the flavonoids. Among the measurable components of phytoestrogens in WWM juice, 8‐prenylnaringenin showed the highest antiviral activity. We synthesized 8‐prenylnaringenin and used liquid chromatography–mass spectrometry to quantitate the active ingredient in WWM. The antiviral activities of 8‐prenylnaringenin were observed against H1N1 and H3N2 influenza A subtypes and influenza B viruses. Moreover, 8‐prenylnaringenin was found to inhibit virus adsorption and late‐stage virus replication, suggesting that the mechanisms of action of 8‐prenylnaringenin may differ from those of amantadine and oseltamivir. We confirmed that 8‐prenylnaringenin strongly inhibited the viral entry of all the influenza virus strains that were examined, including those resistant to the anti‐influenza drugs oseltamivir and amantadine. This result indicates that 8‐prenylnaringenin may activate the host cell's defense mechanisms, rather than directly acting on the influenza virus. Since 8‐prenylnaringenin did not inhibit late‐stage virus replication of oseltamivir‐resistant strains, 8‐prenylnaringenin may interact directly with viral neuraminidase. These results are the first report on the anti‐influenza virus activity of 8‐prenylnaringenin. Our results highlight the potential of WWM and phytoestrogens to develop effective prophylactic and therapeutic approaches to the influenza virus.

## Graphical abstract

The antiviral activities of 8‐prenylnaringenin, which is the ingredient in wild watermelon, were observed against H1N1 and H3N2 influenza subtypes and influenza B viruses, excluding oseltamivir‐resistant H1N1 viruses. Since oseltamivir‐resistant strains were not inhibited by 8‐prenylnaringenin, 8‐prenylnaringenin may interact directly with viral neuraminidase.

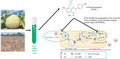

## 1 INTRODUCTION

Influenza is an acute respiratory infection caused by the influenza virus (IFV), which belongs to the family Orthomyxoviridae and it is prevalent worldwide. Types A, B, and C IFV can infect humans with types A and B influenza, causing seasonal epidemics every year and sometimes causing severe complications, such as pneumonia and encephalitis (Morishima, 2002). Vaccines and antiviral drugs are used to prevent and treat IFV infection, respectively. However, these vaccines fail to induce a stable preventive effect (Centers for Disease Control & Prevention, 2020). In addition, the emergence of IFV strains resistant to amantadine and oseltamivir has become a serious problem in recent years (Dapat et al., 2012; Stephenson & Nicholson, 2001). Thus, a novel approach to protect against IFV infection is needed. We hope that we can contribute to reducing the negative effect of resistant influenza virus by improving our diet, taking supplements, and furthering the discovery of small‐molecule drugs.

Recently, functional foods showing antiviral activity have been reported (Chen et al., 2016; Morimoto et al., 2021; Nagai et al., 2018), and ingredients of functional foods have received increased attention. Some foods have been reported to have various ingredients with anti‐INF activity, including tea polyphenols such as catechins, theaflavins, and procyanidins (Yang et al., 2014). Catechins in green tea (Müller & Downard, 2015; Song et al., 2005) showed neuraminidase inhibitory activities and IFV growth inhibitory effect through acidification of the intercellular compartment (Imanishi et al., 2002). In addition, green tea suppressed inflammation, cell proliferation, and apoptosis through the regulation of the nuclear factor kappa B (NF‐κB), an important transcriptional regulator (Di Lorenzo et al., 2013). It has also been suggested that cocoa polyphenols and anthocyanin pigments in hibiscus tea exhibit anti‐IFV activity (Baatartsogt et al., 2016; Kamei et al., 2016). All the components of adlay tea, adlay seeds, naked barley seeds, soybean, and cassia seeds inhibited both IFV adsorption and virus replication, resulting in strong antiviral activity against influenza A H1N1 and H3N2 subtypes and influenza B viruses (Nagai et al., 2018, 2019). The anti‐IFV activity of soybean daidzein differs from that of oseltamivir and functions via signal transduction through 5‐lipoxygenase products (Horio et al., 2020).

*Citrullus lanatus* var. *citroides*, commonly known as wild watermelon (WWM), can adapt and grow under severely dry and high‐ultraviolet light conditions and is native to the Kalahari Desert in southern Africa. In its native region, WWM is used as a dietary source of hydrogen and a water source to wash the body. WWM has a high citrulline content, which protects the plant from the stresses of its native environment (Takahara et al., 2005; Yokota et al., 2002), and the seeds contain many essential amino acids (Umar et al., 2013). Although there have been several reports on the usefulness of WWM, its food functionality remains a relatively new area of research. In a previous study, we reported an anti‐influenza activity of WWM juice, but the effective components remained unknown (Morimoto et al., 2021). In the current study, we aimed to investigate the flavonoid‐based components present in WWM juice and due to the large amount of polyphenols detected, we focused on phytoestrogens, in which daidzein, acacetin, kaempferol, naringenin, and resveratrol have been reported to have anti‐influenza virus effects (Dong et al., 2014; Kim et al., 2001; Nagai et al., 2019; Palamara et al., 2005). It has been hypothesized that the anti‐influenza effect of flavonoids might stem from their ability to coordinate metal ions. We evaluate the activity of prenylated flavonoids against IFV replication. Specifically, we focused on prenylated naringenins because naringenin from *Citrus junos* has been previously shown to inhibit influenza A virus (Kim et al., 2001), and prenylated polyphenols have been shown to accumulate in Caco‐2 intestinal epithelial cells and hepatocytes, with their intracellular concentration being 60 times higher than the extracellular concentration (Wolff et al., 2011). Therefore, this paper examined the antiviral effect of 8‐prenylnaringenin (8‐PN), since we have reported on the antiviral effect of daidzein so far (Horio et al., 2020).

## 2 MATERIALS AND METHODS

### 2.1 Compounds

All reagents used for chemical synthesis not explicitly mentioned were purchased from FUJIFILM Wako Pure Chemical Corporation, Tokyo Chemical Industry Co., Nacalai Tesque, Selleck Biotech, Namiki Shoji Co., Ltd., and Sigma‐Aldrich Co. (±)‐Naringenin was purchased from Cayman Chemical Ltd and dissolved in dimethyl sulfoxide (DMSO) as a stock solution (50 mg/ml). Meanwhile, (±)‐8‐PN was synthesized from (±)‐naringenin in a four‐step process with a 24% overall yield according to a previously reported procedure (Gester et al., 2001) and as detailed in the supplementary methods. In the current study, (±)‐naringenin was used instead of (*S*)‐naringenin, considering the cost.

### 2.2 Cells and viruses

Madin–Darby canine kidney (MDCK) cells were grown in Eagle's minimum essential medium (EMEM; FUJIFILM Wako Pure Chemical Corporation) supplemented with 7% FBS. In the current study, we used influenza A H1N1 strains A/PR/8/34, A/Suita/114/2011, A/Osaka/2024/2009, and A/Osaka/71/2011; H3N2 strains A/Sydney/5/97, and A/Aich/2/68; and B strains B/Shanghai/261/2002 and B/Nagasaki/1/87. Treatment of the cells against viral infections was according to the method by Morimoto et al. (2021). Briefly, the virus culture was diluted in serum‐free MEM containing 0.04% bovine serum albumin (BSA, fraction V; Sigma‐Aldrich) and then incubated with the cells to infect them at a multiplicity of infection (MOI) of 0.001 for 1 h at 37°C. The medium was then removed and replaced with serum‐free DMEM (Dulbecco's modified eagle medium) containing 0.4% BSA and 2 μg/ml acetyl trypsin (Merck Sigma‐Aldrich) for the rest of the infection period.

### 2.3 Metabolomic data analysis

The metabolomic data were obtained via LTQ ORBITRAP XL analysis (Thermo Fisher Scientific) using the Power Get software (http://www.kazusa.or.jp/komics/ja/tool‐ja/48‐powerget.html) originally developed by the Kazusa DNA Research Institute (Ogi et al., 2018). Chromatographic separation was performed at 40°C using a TSK gel ODS‐100V column (3 mm × 50 mm, 5 μm: TOSOH) on an Agilent 1200 series system. For separation, the mobile phases were optima grade water with 0.1% formic acid (A) and acetonitrile with 0.1% formic acid (B). A 25‐min gradient at a flow rate of 0.4 ml/min with the following conditions was used: 0–5 min, held at 1% B; 5–10 min, linear gradient from 1% to 3% B; 10–18 min, linear gradient from 3% to 40% B; 18–22 min, linear gradient from 40% to 80% B; 22–27 min, column cleaning at 95% B; and 27–35 min, re‐equilibration with solution A. The injection volume was 5 µl, and the MS was operated in the positive ion mode (ESI) with a scan range of *m*/*z* 100–1500 using one of the top five MS/MS methods. The average accurate mass of the compound peak was collated with a public database (Flavonoid Viewer) using Kazusa DNA Research Institute development Software (MF Searcher).

### 2.4 LC/MS measurement (triple quadrupole, QQQ)

LC/MS measurement was performed according to a previously described method (Sakurai et al., 2014). Each sample was injected into an LC/MS system comprising an Agilent 1260 Infinity binary LC and an Agilent 6430 triple‐quadrupole LC/MS (Agilent Technologies Inc.). LC parameters were as follows: injection volume: 5 μl; column: Synergi Hydro‐RP 100A (100 mm × 3 mm, φ2.5 μm; Phenomenex Inc.); column oven temperature: 40°C; mobile phase: (A) 2% acetic acid, (B): 0.5% acetic acid/acetonitrile (1:1 v/v); flow rate: 0.4 ml/min. The solvent changes were applied as a linear gradient. Solvent B was increased from 80% to 100% at 6 min, until 8 min, then decreased to 80% until 10 min. MS parameters were as follows: ion source: ESI (negative ion mode); dry gas: nitrogen (350°C, 12 L/min); nebulizing gas: nitrogen (60 psi); capillary voltage: 3500 V; fragmentor voltage: 110 V; and multiple reaction monitoring (MRM) mode (precursor ion: *m/z* 339.1, product ion: *m/z* 219.1, collision energy: 14 V). Analysis was carried out according to a previously described method (Kammerer et al., 2004).

### 2.5 Cell viability determination

Cell viability was evaluated using a Cell Proliferation Kit I (MTT), according to the manufacturer's instructions (F. Hoffmann–La Roche Ltd). Briefly, MDCK cells were monophasically cultured on a 96‐well flat bottom plate and then washed twice with serum‐free MEM. Samples were added to DMEM containing 2 μg/ml acetyl trypsin and 0.4% BSA, and a 100‐µl sample of each serial dilution was added to each well. Then, the cells were cultured in a CO_2_ incubator at 37°C for 24 h. After culturing, MTT standard reagent was added 10 µl/well, and the cells were cultured in a CO_2_ incubator for 4 h. Subsequently, 100 µl of the solubilized solution was added to the each well, and the cells were cultured in a CO_2_ incubator overnight. The complete solubilization of purple forma remnants was checked and the absorbance was measured using a microplate reader (TECAN Infinite M200) at a wavelength of 575 nm and a reference wavelength of 650 nm.

### 2.6 Antiviral assay of 8‐PN

The effects of the addition of the compounds on viral yield were determined as previously described (Nagai et al., 2018), with slight modifications. MDCK cells were cultured in 24‐well plates (Thermo Fisher Scientific) at 1 × 10^5^ cells/well in 500 μl/well of EMEM containing FBS and incubated for 24 h at 37°C. In case of adsorption inhibition, diluted viruses were allowed to infect confluent cells at an MOI of 0.01 for 1 h at 37°C with or without 11.4 µg/ml 8‐PN. After 1 h of adsorption, infected cells were rinsed once with serum‐free EMEM and then cultured in DMEM supplemented with 0.4% bovine serum albumin (BSA, fraction V; Sigma‐Aldrich; 500 µl/well) without 8‐PN. After 8 h, the infected cells as IFV samples were frozen at −80°C and subjected to two freeze‐thaw cycles prior to determining the viral yield by focus‐forming assays. In the case of replication inhibition, diluted viruses were allowed to infect the cells at an MOI of 0.001 for 1 h at 37°C. After 1 h of adsorption, the infected cells were rinsed once with serum‐free EMEM and then cultured in DMEM containing 0.4% BSA (500 µl/well) with or without 11.4 µg/ml 8‐PN. After 24 h, the supernatants were collected as IFV samples and subjected to focus‐forming assays.

### 2.7 Focus‐forming reduction assay for measuring virus titer

Focus‐forming reduction assay (FFRA) was performed as previously described (Morimoto et al., 2021; Nagai et al., 2018). In brief, infected cells were visualized by adding murine monoclonal anti‐HA antibody (C179 for H1, F49 for H3, and 7B11 for B; Morimoto et al., 2021) and a goat anti‐mouse IgG antibody conjugated to horseradish peroxidase (Merck KGaA).

### 2.8 Time‐of‐addition assay

**FIGURE 1:**
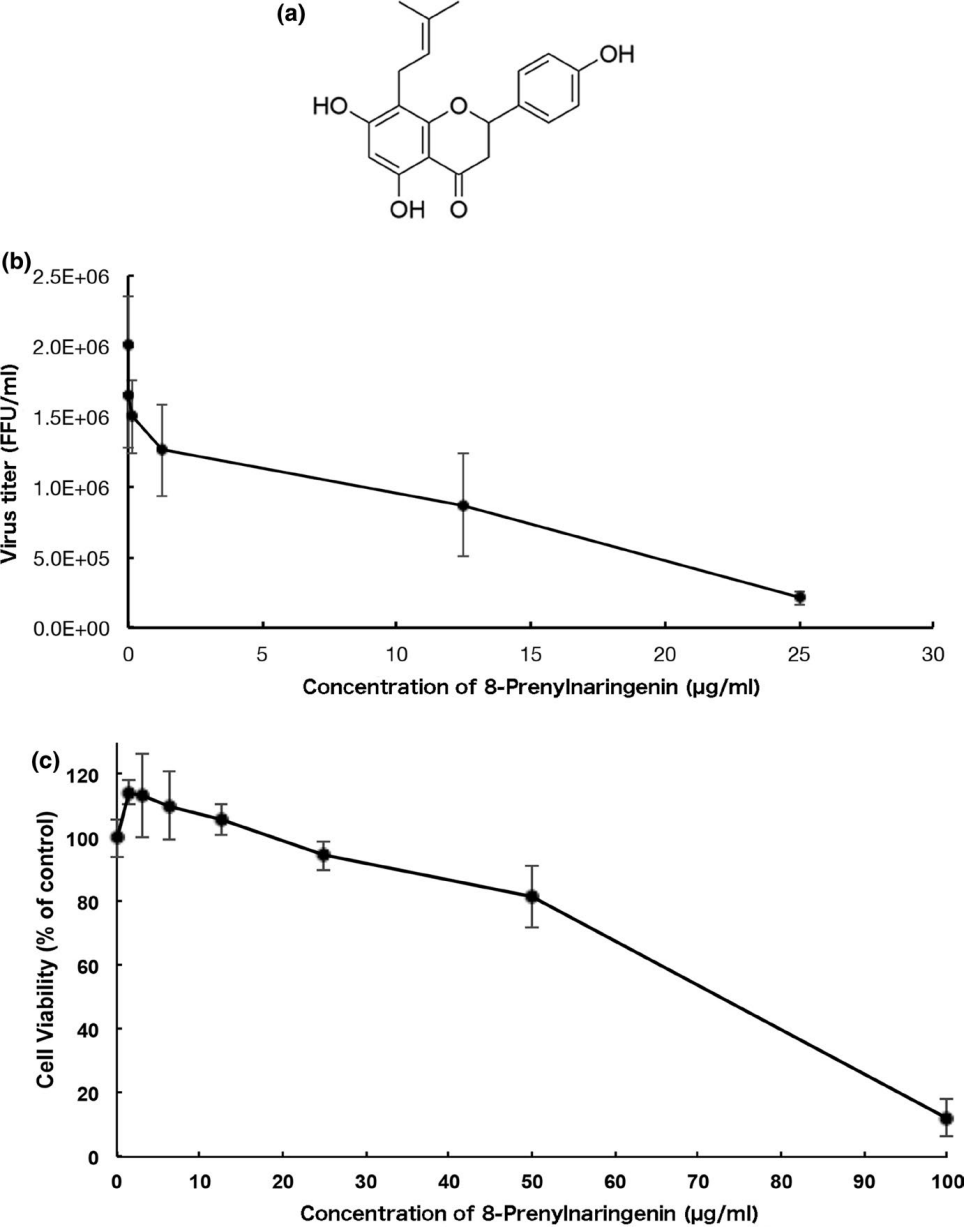
8‐PN inhibited H1N1 influenza virus replication in MDCK cells. MDCK cells were incubated with influenza A/PR/8/34 virus at a multiplicity of infection of 0.001. Viral yields were determined 24 h postinfection using focus‐forming assays. Vertical lines indicate standard deviation (*n* = 3). (a) Structure of 8‐PN. (b) Concentration‐dependent inhibitory effect of 8‐PN compounds on virus multiplication. The virus titer was determined 24 h postinfection using focus‐forming assays. (c) Cytotoxicity of 8‐PN compounds. Vertical lines indicate standard deviations (*n* = 3). Data are representative of three independent experiments. FFU is focus‐forming unit

**FIGURE 2:**
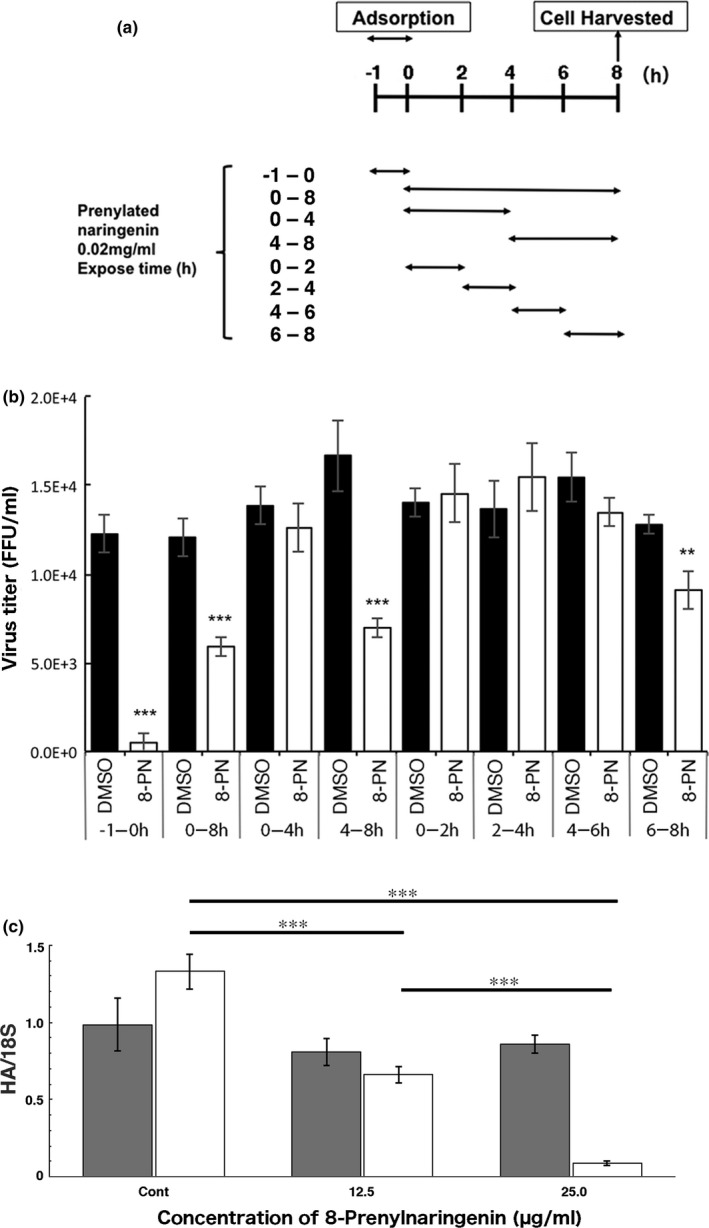
Effect of 8‐prenylnaringenin (8‐PN) on viral replication. MDCK cells in 24‐well plates were infected with influenza virus A/PR/8/34 at a multiplicity of infection of 0.01. DMEM containing 8‐PN (0.02 mg/ml: more than twice the IC_50_ value) was then added at the different times shown. (a) Time‐of‐addition assay schedule. (b) After infection according to the indicated schedule, the cells were harvested, and viruses were assayed using focus‐forming reduction assays. Filled columns, mean viral yields of control cells; open columns, mean viral yields of cells treated with 8‐PN. Data are representative of three independent experiments. (c) Effect of 8‐PN on viral binding to cells. Closed columns indicate viral adsorption onto cells at 4°C at three different concentrations of 8‐PN; open columns, 37°C. Error bars indicate SD (*n* = 3). Data are representative of two independent experiments. *p < .05; **p < .01; ***p < .001. DMSO, dimethyl sulfoxide; FFU, focus‐forming unit; HA, HA protein

We conducted a time‐of‐addition experiment using a previously described procedure (Morimoto et al., 2021) with slight modifications. The difference was the concentration of the inhibitor, 8‐PN. DMEM containing 0.02 mg/ml of the compounds, which was approximately 80% the maximum inhibitory concentration (Figure 1), was added at different periods of infection: during adsorption, for 1 h incubation with viruses; during replication for up to 8 h, measured every two‐ and four‐hour intervals (Figure 2a). The infected cells were then frozen at −80°C 8 h after infection and subjected to two freeze‐thaw cycles before determining the viral yield using the focus‐forming assay.

### 2.9 Viral binding inhibition assay

The viral amount attached to the cells was determined by measuring the viral RNA encoding the HA protein (HA) using SYBR green and a pair of primers, HA‐F: 5′‐TTGCTAAAACCCGGAGACAC and HA‐R: 5′‐CCTGACGTATTTGGGCACT. Viral RNA bound to cells was extracted, and cDNA was synthesized; viral RNA was quantified as described previously (Nagai et al., 2018). As a normalization gene for real‐time PCR based on influenza virus‐infected cells, 18S rRNA was quantified as described previously (Kuchipudi et al., 2012).

## 3 RESULTS

### 3.1 Metabolomic data analysis of WWM juice

We conducted a metabolomic analysis to identify the active components in WWM juice, focusing on flavonoids that have been reported. Many low‐molecular weight compounds were identified (1646), including 578 different flavonoids that comprised 35% of the total compounds present in the WWM juice (Table 1). There were 228 glycosylated flavonoids and 350 aglycons. Thus, the proportion of aglycons compared to all flavonoids detected was 61%. The WWM juice contained 173 prenylated flavonoids, which accounted for 30% of the detected flavonoids, and 172 of the prenylated flavonoids were aglycons. Some of the prenylated flavonoids detected are shown in Table 2. In the metabolome analysis, 8‐PN and 8‐prenyldaidzein were detected in WWM juice, while daidzein, genistein, biochanin, kaempferol derivatives, secoisolariciresinol, pinoresinol, and glycosylated variants of the latter phytoestrogens were also present. However, acacetin, kaempferol, resveratrol, glycitein, formononetin, coumestrol, 4‐methoxycoumesterol, repensol, trifoliol, and lariciresinol were not detected (Table S1).

**TABLE 1:** Classifying polyphenols in WWM juice based on backbone structure

Backbone name and structure	Molecular weight	Numbers	%
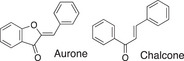	222, 208	83	14
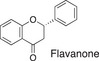	224	99	17
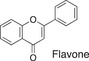	222	182	31
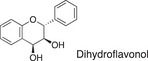	242	23	4
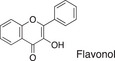	238	90	16
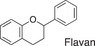	210	6	1
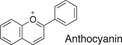	207	0	0
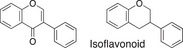	222, 210	91	16
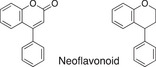	222, 210	4	1

**TABLE 2:** Classifying polyphenols based on ornamentation groups

Glycosylation	Modification	Numbers
No	No	91
No	Alkylated	12
No	Prenylated	172
No	possessing a furan group	22
No	possessing a pyran group	53
O‐glycoside	No	120
O‐glycoside	Alkylated	1
O‐glycoside	Prenylated	1
O‐glycoside	possessing a furan group	1
O‐glycoside	Phenylpropanoid	8
C‐glycoside	No	62
O‐ & C‐glycoside	No	18
Others		17

### 3.2 Quantitation of 8‐PN and other phytoestrogens in WWM juice

The antiviral activity of one of the prenylated flavonoids, 8‐PN, was measured, and the results are summarized in Table 1. We focused on prenylated naringenins, such as 8‐PN (Figure 1a), which was detected by liquid chromatography–mass spectrometry measurement (QQQ) at approximately 0.53 ng/ml in the WWM juice. The chemically synthesized 8‐PN strongly inhibited viral growth in MDCK cells (Figure 1b). Moreover, it showed no evidence of cytotoxicity at concentrations lower than 25 µg/ml (Figure 1c). We then evaluated viral replication in MDCK cells treated with naringenin and 8‐PN. Both naringenin and 8‐PN inhibited IFV growth in a concentration‐dependent manner, but the virus growth inhibition activity of 8‐PN was approximately 13 times higher than that of naringenin (Table S2). The IC_50_ values of naringenin and 8‐PN were 70 and 5.5 μg/ml, respectively. Acacetin and daidzein derivatives detected in WWM juice by QQQ, but kaempferol and resveratrol were not detected. The IC_50_ values of acacetin was 9.6 μg/ml and acacetin was detected at approximately 0.86 ng/ml in the WWM juice. The IC_50_ values of daidzein was 28 μg/ml. Since 8‐prenyldaidzein, a daidzein derivative, is not available in Japan, neither its antiviral activity nor its concentration in WWM juice could be measured. Another daidzein derivative was glycosylated daidzein, which did not have antiviral activity in vitro. Daidzin, a glycosylated daidzein, and astragalin, Kaempferol‐3‐O‐glucoside, were not quantitative detection in the WWM juice, and it was not possible to measure the IC_50_ values of daidzin and astragalin. The antiviral activity of glycitin, glycosylated glycitein, was much weaker than that of aglycone glycitein (unpublished data). Genistein, biochanin, and those derivatives also did not have antiviral activity in vitro. The IC_50_ values of (+)‐pinoresinol was 123 μg/ml and it was detected by QQQ at approximately 80.17 ng/ml in the WWM juice. Glycosylated secoisolariciresinol (secoisolariciresinol diglucoside) and pinoresinol (pinoresinol diglucoside) had antiviral activities in vitro. The IC_50_ values of pinoresinol diglucoside and secoisolariciresinol diglucoside were 55 and 44 µg/ml, respectively. Pinoresinol diglucoside was contained at a concentration of approximately 42.63 ng/ml and secoisolariciresinol diglucoside was not quantitatively detected in WWM juice (Table S2).

### 3.3 The critical steps targeted by 8‐PN

The stage of viral replication inhibited by 8‐PN was identified using time‐of‐addition assays. Figure 2a shows the periods at which 8‐PN was included in the incubation mixture. As reported previously (Nagai et al., 2018), one replication cycle of A/PR/8/34 within a cell takes approximately 8 h. Based on this information, the stages of viral multiplication blocked by 8‐PN were elucidated, as demonstrated in Figure 2b. We then segmented the exposure period to 8‐PN during viral replication into 2‐h intervals. The results revealed that two different steps of the virus infection process were inhibited by 8‐PN. The first step was viral adsorption to the cells (−1 to 0 h of viral infection). The second step was during actual viral replication, specifically the late stage of replication (4–8 h postinfection), and especially, the period associated with viral assembly (6–8 h postinfection). Thus, the time‐of‐addition assay showed that 8‐PN blocks at least two stages of viral growth, adsorption and late replication (Figure 2b). On the other hand, like daidzein, 8‐prenyldaidzein is expected to inhibit virus maturation rather than inhibit virus adsorption.

### 3.4 Viral adsorption inhibition by 8‐PN

8‐PN restricted viral entry in all type A and type B IFVs evaluated, including oseltamivir‐resistant viruses such as A/Osaka/2024/2009 and A/Osaka/71/2011 (Table 3). This suggested that the mechanism of action of the ingredients in WWM may differ from that of amantadine (Stephenson & Nicholson, 2001). A/Osaka/2024/2009 and A/Osaka/71/2011, which are H1N1 2009 pandemic (pdm09) viruses, and type B viruses are amantadine resistant (Dapat et al., 2012; Stephenson & Nicholson, 2001).

**TABLE 3:** Effect of 8‐prenylnaringenin on the multiplication of various influenza virus strains

Virus type and strain	Adsorption		Replication	
	IC_50_ ± SD (µg/ml)	SI^a^	IC_50_ ± SD (µg/ml)	SI^a^
A (H1N1)				
PR/8/34	18.4 ± 3.1	3.6	5.5 ± 0.4	12.2
Suita/114/2011	12.3 ± 2.6	5.4	24.3 ± 1.1	2.8
Osaka/2024/2009^b^	6.4 ± 2.1	10.5	ND	ND
Osaka/71/2011^b^	14.3 ± 2.1	4.7	ND	ND
A (H3N2)				
Sydney/5/97	7.1 ± 0.7	9.4	2.9 ± 1.6	23.1
Aich/2/68	6.7 ± 0.6	10.0	2.5 ± 0.2	26.8
B				
Shanghai/261/2002	11.7 ± 0.3	5.7	2.2 ± 0.4	30.4
Nagasaki/1/87	12.3 ± 0.2	5.4	6.6 ± 0.2	10.1

Abbreviation: ND, not detected.

a Selectivity index = CC_50_/IC_50_, CC_50_ = 66.9 µg/ml.

b Oseltamivir‐resistant virus, pdm09.

The addition of 8‐PN inhibited viral adsorption (Table 3) in a temperature‐ and concentration‐dependent manner (Figure 2c), indicating that 8‐PN affects viral endocytosis. The mechanism of adsorption inhibition was not due to the interaction between 8‐PN and viral components but may have resulted from the interaction between 8‐PN and cell components via signal transduction, similar to that of daidzein and flavonoids (Dong et al., 2014; Horio et al., 2020).

### 3.5 Viral replication inhibition by 8‐PN

Regarding the inhibition of replication, 8‐PN inhibited all type A and type B IFV, except for oseltamivir‐resistant viruses, such as A/Osaka/2024/2009 and A/Osaka/71/2011 (Table 3). This implies that the mechanism of action of 8‐PN may be the same as that of oseltamivir.

Meanwhile, the inhibition of late replication by 8‐PN may have been associated with viral neuraminidase as 8‐PN did not inhibit the replication of oseltamivir‐resistant viruses (Table 3). Therefore, the mechanism underlying 8‐PN viral replication inhibition may be the interaction between viral neuraminidase and 8‐PN, that is, the direct inhibition of neuraminidase by 8‐PN, similar to oseltamivir.

## 4 DISCUSSION

Due to the large amount of polyphenols detected, we first focused on phytoestrogens, in which daidzein, acacetin, kaempferol, naringenin, and resveratrol have been reported to have anti‐influenza virus effects (Dong et al., 2014; Kim et al., 2001; Nagai et al., 2019; Palamara et al., 2005), and the details of their inhibition mechanisms of daidzein, kaempferol, and resveratrol have also been investigated (Dong et al., 2014; Horio et al., 2020; Palamara et al., 2005). However, phytoestrogens other than genistein (which was reported to have no anti‐influenza virus effects; Nagai et al., 2019), like 8‐PN, have not had their antiviral activity reported. In the metabolome analysis, the naringenin derivative 8‐PN, genistein derivatives, and daidzein derivatives were detected in WWM juice, but acacetin, kaempferol, and resveratrol were not detected. However, acacetin was detected by QQQ and showed about half the levels of antiviral activity of 8‐PN. This time, we could not measure the antiviral activity of 8‐prenyldaidzein, but the relationship between naringenin and 8‐PN suggests that the antiviral activity of 8‐prenyldaidzein may be 10 times higher than that of daidzein. It may have an even stronger activity than 8‐PN, but its inhibitory mechanism is predicted to be very similar to that of daidzein shown by Horio et al. (2020). The phytoestrogen 8‐prenyldaidzein may also play an important role as an inhibitor in the late stages of viral growth. A phytoestrogen, (+)‐pinoresinol, showed a 22 times lower antiviral activity than 8‐PN. However, no, or very weak, antiviral activities were observed in glycosylated flavonoid of phytoestrogens. Although the antiviral activity of pinoresinol diglucoside increased about three‐fold against pinoresinol aglycon, pinoresinol diglucoside showed a 10 times lower antiviral activity than 8‐PN. Therefore, this paper examined the antiviral effect of 8‐PN. Like various polyphenols, naringenin also shows anti‐influenza activity (Horio et al., 2020; Kim et al., 2001; Nagai et al., 2019; Zima et al., 2020). While Zn regulated the influenza virus replication (Nakashima et al., 2021), it has been suggested that primate cells such as Vero‐E6 cells require ionophores for zinc uptake (Te Velthuis et al., 2010). It was hypothesized that the anti‐influenza effect of flavonoids might stem from their ability to coordinate metal ions, as documented by various quercetin‐metal ion complexes reported in the literature (Liu & Guo, 2015; Torreggiani et al., 2005). Prenylation of polyphenols not only creates a new affinity for membranes (Eesolowska et al., 2014) but may also affect permeability. In cell experiments, there is also a report that a prenylated polyphenol, xanthohumol, is concentrated 60‐fold in cells (Wolff et al., 2011). In addition, the prenylated polyphenol is bound to cellular proteins, which may alter the properties of cellular factors (Wolff et al., 2011). Thus, it has been suggested that prenylated polyphenols may be involved in intracellular signal transduction and enzymatic and physiological activities. Furthermore, a wide range of bioactivities, such as the prevention of osteoporosis and anticancer activities, are known for prenylated polyphenols, such as 8‐PN (Štulíková et al., 2018). Daidzein, known as phytoestrogen, exhibited anti‐influenza activity by activating cells at the late replication stage (Horio et al., 2020), but this is the first report on anti‐influenza activity of 8‐PN on the two stages. Notably, the mechanisms of action of daidzein and 8‐PN were found to be different.

The time‐of‐addition assay (Figure 2b) showed that 8‐PN blocks at least two stages of viral growth, adsorption and late replication. Regarding adsorption inhibition, 8‐PN restricted viral entry of all type A and type B IFVs evaluated, including amantadine‐ and oseltamivir‐resistant viruses (Table 3). H1N1 2009 pandemic (pdm09) viruses and type B viruses are amantadine resistant (Dapat et al., 2012; Stephenson & Nicholson, 2001). This suggested that the mechanism of action of the WWM ingredients may differ from that of amantadine (Stephenson & Nicholson, 2001). IFVs are internalized via receptor‐mediated endocytosis (Laladamyali et al., 2004), and inhibition of endocytosis may effectively prevent infection. For instance, resveratrol, a natural polyphenol, reduces the internalization of cholera toxin by inhibiting its endocytosis into the cells (Morinaga et al., 2010). As adsorption inhibition by 8‐PN was shown to be temperature dependent, 8‐PN may also exert its activity by inhibiting endocytosis of IFV.

Regarding replication inhibition, 8‐PN inhibited all type A and type B IFVs, except for oseltamivir‐resistant viruses such as A/Osaka/2024/2009 and A/Osaka/71/2011 (Table 3). This implies that the mechanism of action of 8‐PN may be the same as that of oseltamivir. Although 8‐PN failed to show antiviral activity against oseltamivir‐resistant viruses, WWM juice exhibits antiviral activity against these viruses (Morimoto et al., 2021). This suggests that WWM contains additional ingredient(s) with antiviral activities that affect the replication of oseltamivir‐resistant viruses, similar to the activity of daidzein (Horio et al., 2020).

Since phytoestrogens have high antiviral activity, this study revealed that phytoestrogens were present in WWM juice and showed that naringenin became 10‐fold more active by prenylation. Prenylation increases the antiviral activity of polyphenols, which facilitates intracellular uptake and may have facilitated accumulation in the cells (Eesolowska et al., 2014; Wolff et al., 2011). While glycosylation may make flavonoids of phytoestrogens water soluble, it also may result in the reduction or loss of antiviral activity due to in vitro membrane permeation difficulty. Glycosides of phytoestrogens other than flavonoids had higher antiviral activity than the aglycones, suggesting that there may be at least two groups with different signaling pathways. The current study has shown a hitherto unknown anti‐IFV activity of 8‐PN. However, because the levels of 8‐PN in WWM are inadequate to exert the observed antiviral activity, other antiviral ingredients were likely involved. As the antiviral effect of WWM is probably a combined effect of several ingredients, further studies are needed to identify the other active ingredients and establish the precise mechanisms of action.

This study evaluated the anti‐IFV activity of the ingredients of WWM, which were detected by metabolome analysis, and demonstrated antiviral activity by 8‐PN. The ingredient(s) inhibited the viral adsorption and late replication stages in the growth process of IFVs. Our results also indicate that the antiviral mechanism of 8‐PN against IFV growth during virus adsorption may differ from that of amantadine, while the mechanism of endocytosis and late replication inhibition may be similar to that of oseltamivir. This is the first report of the anti‐IFV action of 8‐PN. Furthermore, the study findings highlight the potential role of WWM in the development of novel prophylactic and therapeutic approaches against influenza.

## CONFLICT OF INTEREST

Ayaka Nakashima, Taro Ogawa and Kengo Suzuki are employees of euglena Co., Ltd. All other authors declare no competing interests.

## ETHICAL APPROVAL

This study does not involve any human or animal testing.

## Supporting information

Supplementary Material

Table S1

Table S2

## Data Availability

The datasets analyzed during the current study are available from the corresponding author on reasonable request.
