# Supplementary material for: Influenza virus entry and replication inhibited by 8‐prenylnaringenin from Citrullus lanatus var. citroides (wild watermelon)
Source: Food Sci Nutr. 2022 Jan 23;10(3):926–35. doi: 10.1002/fsn3.2725 (PMC8907720; doi:10.1002/fsn3.2725)
Supplement: Supplementary file 1 — Supplementary Material [file FSN3-10-926-s003.docx]

**Supplemental methods**

**Synthesis of (±)-8-Prenylnaringenin**

**General**

All materials for chemical synthesis not explicitly mentioned were purchased from Wako Pure Chemical Products Co. (Osaka, Japan), Tokyo Kasei Kogyo Co. (Tokyo, Japan), Nacalai Tesque Co. (Kyoto, Japan), and Aldrich Chemical Co. (USA). (±)-Naringenin was purchased from Cayman chemical Ltd., and was used directly without purification. ^1^H NMR (500 MHz) was recorded on a JEOL JNM-ECP500 spectrometer. Chemical shift values were expressed in ppm relative to a solvent residual signal of CDCl_3_ (7.26 ppm), DMSO-*d6* (2.50 ppm) in ^1^H NMR. Abbreviations are as follows: s, singlet; d, doublet; t, triplet; m, multiplet; br, broad. Coupling constant (*J*) values were expressed in Hz. Flash column chromatography was performed with silica gel (Wakosil C-200) obtained from Wako Pure Chemical Products Co. Analytical thin layer chromatography (TLC) was performed on Merck Silica gel 60 F_254_ glass plates and visualization was accomplished with a UV lamp.

**Naringenin-4’,7-diacetate (2)**

According to the reported procedure [S1], to a solution of (±)-naringenin (**1**) (5.44 g, 20.0 mmol) in 20.0 mL of anhydrous pyridine was added dropwise acetic anhydride (4.08 g, 40.0 mmol) at room temperature, and then stirred for overnight. The resulting suspension was poured into ice water, and the precipitate was collected by filtration, washed twice with water and recrystallized from methanol to give 5.92 g (83%) of titled compound as colorless needles. ^1^H NMR (DMSO-*d6*) δ 2.26 (s, 3H), 2.28 (s, 3H), 2.90 (dd, 1H, *J* = 2.8, 17.2), 3.46 (dd, 1H, *J* = 13.3, 17.2), 5.73 (dd, 1H, *J* = 2.8, 13.3), 6.37 (d, 1H, *J* = 2.3), 6.39 (d, 1H, *J* = 2.3), 7.21 (d, 2H, *J* = 8.3), 7.59 (d, 2H, *J* = 8.3), 11.93 (s, 1H).

**2-(4-Acetoxyphenyl)-5-(3-methylbut-2-enyloxy)-4-oxochroman-7-yl acetate (3)**

According to the reported procedure [S1], to a solution of **2** (3.56 g, 10 mmol) and triphenylphosphine (3.20 g, 12.2 mmol) in 120 mL of anhydrous THF (with BHT as a stabilizer) was added prenyl alcohol (1.29 g, 15.0 mmol) under an argon atmosphere. A solution of diethyl azodicarboxylate (2.78 g, 16.0 mmol) in 37.5 mL of anhydrous THF (with BHT as a stabilizer) was slowly added to the mixture over 2 h using syringe pump under ice water bath cooling, and then stirred for 2 h at room temperature. After removal of the solvent by evaporation, the crude product was purified by flash column chromatography eluting with ethyl acetate and hexane in 1/3 to 1/2 to give 3.50 g of white solid. The solid was recrystallized from ethyl acetate and hexane gave 2.51 g of titled compound as a white powder. ^1^H NMR (CDCl_3_) δ 1.74 (s, 3H), 1.79 (s, 3H), 2.30 (s, 3H), 2.31 (s, 3H), 2.82 (dd, 1H, *J* = 3.2, 16.5), 3.02 (dd, 1H, *J* = 13.2, 16.5), 4.61 (br-s, 2H), 5.43 (dd, 1H, *J* = 3.2, 13.2), 5.52–5.55 (m, 1H), 6.32 (d, 1H, *J* = 2.3), 6.43 (d, 1H, *J* = 2.3), 7.15 (d, 2H, *J* = 8.7), 7.47 (d, 2H, *J* = 8.7).

**2-(4-Acetoxyphenyl)-5-hydroxy-8-(3-methylbut-2-enyl)-4-oxochroman-7-yl acetate (4)**

**2-(4-Acetoxyphenyl)-6-(1,1-dimethylallyl)-5-hydroxy-4-oxochroman-7-yl acetate (5)**

According to the reported procedure [S1], a solution of **3** (3.50 g, 8.25 mmol) and Eu(fod)_3_ (868 mg, 0.837 mmol) in 1.40 mL of dry CHCl_3_ was stirred at 60 °C for 12 h under an argon atmosphere. After complete consumption of the starting material which was checked by TLC, the reaction mixture was concentrated in vacuo, and the residue was purified by flash column chromatography eluting with ethyl acetate and hexane in 1/3 ratio to give 3.34 g of **4** which include a small amount of **5** as a pale yellow solid. The compound **4** was used directly for the next step without further purification.

**(±)-8-Prenylnaringenin (8-PN)**

According to the reported procedure [S1], to a solution of a mixture of **4** and **5** (3.34 g, 7.88 mmol) in 82.0 mL of methanol was added K_2_CO_3_ (163 mg, 1.18 mmol) and 0.20 mL of water. The mixture was heated at 40 °C for 4 h. After complete consumption of the starting material which was checked by TLC, the reaction mixture was concentrated in vacuo. 10.0 mL of satd. aqueous NaHCO_3_ was added to the residue, and the mixture was extracted twice with CH_2_Cl_2_. The combined organic layer was dried over Na_2_SO_4_, filtered, and concentrated in vacuo. The crude products were purified by flash column chromatography eluting with methanol and CH_2_Cl_2_ in 1/20 ratio to give pale yellow solid, and the separated component was recrystallized from CHCl_3_ to give 1.20 g (48%, 2 steps from **3**) of titled compound as a pale yellow solid. ^1^H NMR (DMSO-*d6*) δ 1.54 (s, 3H), 1.59 (s, 3H), 2.72 (dd, 1H, *J* = 3.2, 17.0), 3.08 (d, 2H, *J* = 7.3), 3.21 (dd, 1H, *J* = 12.8, 17.0), 5.09 (t, 1H, *J* = 7.3), 5.43 (dd, 1H, *J* = 3.2, 12.8), 5.97 (s, 1H), 6.80 (d, 2H, *J* = 8.3), 7.32 (d, 2H, *J* = 8.3), 9.58 (s, 1H), 10.76 (br-s, 1H), 12.12 (s, 1H).

**References**

S1. Gester S, Metz P, Zierau O, et al (2001) An efficient synthesis of the potent phytoestrogens 8-prenylnaringenin and 6-(1,1-dimethylallyl)naringenin by europium(III)-catalyzed Claisen rearrangement. Tetrahedron 57:1015–1018.
