# Supplementary material for: Influenza virus entry and replication inhibited by 8‐prenylnaringenin from Citrullus lanatus var. citroides (wild watermelon)
Source: Food Sci Nutr. 2022 Jan 23;10(3):926–35. doi: 10.1002/fsn3.2725 (PMC8907720; doi:10.1002/fsn3.2725)
Supplement: Supplementary file 2 — Table S1 [file FSN3-10-926-s001.docx]

Supplemental Table S1. Phytoestrogens in WWM juice by the metabolomic analysis

Phytoestrogen Modification Modification Modification

None Prenylated Glycosylated

Daidzein × ○ ○

Genistein × × ○

Biochanin × × ○

Glycitein × × ×

Naringenin × ○ ×

Acacetin × × ×

Kaempferol × × ○

Secoisolariciresinol × × ○

Pinoresinol ○ × ○

Resveratrol × × ×

Formononetin × × ×

Coumestrol × × ×

4-Methoxycoumesterol × × ×

Repensol × × ×

Trifoliol × × ×

Lariciresinol × × ×

○：detection; ×:not detection
