# Supplementary material for: Influenza virus entry and replication inhibited by 8‐prenylnaringenin from Citrullus lanatus var. citroides (wild watermelon)
Source: Food Sci Nutr. 2022 Jan 23;10(3):926–35. doi: 10.1002/fsn3.2725 (PMC8907720; doi:10.1002/fsn3.2725)
Supplement: Supplementary file 3 — Table S2 [file FSN3-10-926-s002.docx]

Supplemental Table S2. Antiviral activities and concentrations of phytoestrogens in WWM juice by QQQ

Phytoestrogen Modification Modification Modification

None Prenylated Glycosylated

IC_50_ Conc. IC_50_ Conc. IC_50_ Conc.

Daidzein 28 ND – – ND NQD

Genistein ND ND – – ND –

Biochanin ND ND – – ND –

Glycitein – ND – – – ND

Naringenin 70 ND 5.5 0.53 ­– –

Acacetin 9.6 0.86 – – – –

Kaempferol 57 ND – – ND –

Secoisolariciresinol – ND – – 55 NQD

Pinoresinol 123 80.17 – – 44 42.63

Resveratrol – ND – – – –

Formononetin – – – – – –

Coumestrol – – – – – –

4-Methoxycoumesterol – – – – – –

Repensol – – – – – –

Trifoliol – – – – – –

Lariciresinol – – – – – –

IC_50_: µg/mL; Conc. is concentration: ng/mL; ND: not detected; NQD: no quantitative detection; –: not analyzed
